# Examining differences between overweight women and men in 12-month weight loss study comparing healthy low-carbohydrate vs. low-fat diets

**DOI:** 10.1038/s41366-020-00708-y

**Published:** 2020-11-14

**Authors:** Lucia Aronica, Joseph Rigdon, Lisa C. Offringa, Marcia L. Stefanick, Christopher D. Gardner

**Affiliations:** 1 Department of Medicine, Stanford Prevention Research Center, Stanford University School of Medicine, Stanford, CA USA; 2 Department of Nutritional Sciences, University of Vienna, Vienna, Austria; 3 Quantitative Sciences Unit, Stanford University School of Medicine, Stanford, CA USA

**Keywords:** Nutrition, Randomized controlled trials

## Abstract

**Background/objectives:**

Biological sex factors and sociocultural gender norms affect the physiology and behavior of weight loss. However, most diet intervention studies do not report outcomes by sex, thereby impeding reproducibility. The objectives of this study were to compare 12-month changes in body weight and composition in groups defined by diet and sex, and adherence to a healthy low carbohydrate (HLC) vs. healthy low fat (HLF) diet.

**Participants/methods:**

This was a secondary analysis of the DIETFITS trial, in which 609 overweight/obese nondiabetic participants (age, 18–50 years) were randomized to a 12-month HLC (*n* = 304) or HLF (*n* = 305) diet. Our first aim concerned comparisons in 12-month changes in weight, fat mass, and lean mass by group with appropriate adjustment for potential confounders. The second aim was to assess whether or not adherence differed by diet-sex group (HLC women *n* = 179, HLC men *n* = 125, HLF women *n* = 167, HLF men *n* = 138).

**Results:**

12-month changes in weight (*p* < 0.001) were different by group. HLC produced significantly greater weight loss, as well as greater loss of both fat mass and lean mass, than HLF among men [−2.98 kg (−4.47, −1.50); *P* < 0.001], but not among women. Men were more adherent to HLC than women (*p* = 0.02). Weight loss estimates within group remained similar after adjusting for adherence, suggesting adherence was not a mediator.

**Conclusions:**

By reporting outcomes by sex significant weight loss differences were identified between HLC and HLF, which were not recognized in the original primary analysis. These findings highlight the need to consider sex in the design, analysis, and reporting of diet trials.

## Introduction

As precision medicine is gaining traction, the NIH has highlighted the need to consider sex as a biological variable (SABV) in animal and human research [1–3]. Diet interventions remain the primary strategy in obesity management; yet, their effectiveness is highly variable in the general population and among research participants [4]. Some of this variability reflects biological “sex” differences, such as body composition and metabolism, and some reflects sociocultural “gender” factors, which can influence food behaviors, such as dietary preferences and adherence. In terms of biological sex differences, men tend to lose more weight on a diet intervention because, on average, they have greater body size, higher muscle-to-fat mass ratio, and higher resting and total energy expenditure. They also tend to accumulate more intra-abdominal fat than women, which is associated with higher risk of metabolic syndrome (MetS) and better response to low-carbohydrate diets [5–9]. In terms of behavioral gender differences, women are more likely to attempt weight loss multiple times and join weight loss studies than men [10–12]. They also express a higher preference for low-fat (LF) products and a higher concern towards high-fat foods [12], which might make it easier for them to adhere to a LF diet.

Because most weight loss trials have a much higher representation of women than men, only a few trials have had sufficient power to compare the effects of caloric restriction on weight loss or body composition changes in women vs. men [13, 14], particularly with respect to effects of diets of different macronutrient composition on total weight loss [9, 15–19] or fat and/or lean mass changes [9, 15, 17, 20–23]. In addition, most of these trials had small sample sizes (fewer than 100 participants) and/or were of short duration (less than 6 months). While these studies generally reported greater absolute weight loss for women than for men, these differences were often not significant when adjusted for baseline weight [24, 25]. In a previous study, when sex was not considered, adherence was comparable on a low-fat vs. low-carb diet, but was associated with greater weight loss only for the low carb group [26].

To address this gap in the literature, a secondary analysis of the Diet Intervention Examining The Factors Interacting with Treatment Success trial (DIETFITS) was conducted. DIETFITS involved 609 overweight/obese non-diabetic participants (age, 18–50 years) that were randomized to a 12-month healthy low-carbohydrate (HLC) (*n* = 304) or healthy LF (HLF) (*n* = 305) diet [27]. The first aim of this study was to compare 12-month changes in body weight, fat mass, and lean mass in women and men assigned to HLC or HLF (HLC women *n* = 179, HLC men *n* = 125, HLF women *n* = 167, HLF men *n* = 138). The second aim was to assess whether or not adherence differed by diet-sex group.

## Participants and methods

### Study design and participants

The original DIETFITS trial was a single-site, parallel-group, randomized trial of 609 overweight/obese women (*n* = 346) and men (*n* = 263) conducted at the Stanford Prevention Research Center from January 2013 to May 2016 and was designed to test whether baseline genetic or metabolic factors would explain any of the differential weight loss for participants assigned to HLF vs. HLC [27]. The detailed primary study protocol has been reported elsewhere [28]. Briefly, participants were 609 generally healthy adults assigned to HLC (*n* = 179 women, *n* = 125 men) or HLF (*n* = 167 women, *n* = 138 men) aged 18–50 years, with body mass indices 28–40 kg/m^2^. Exclusion criteria included uncontrolled metabolic disease or hypertension; pregnancy or lactation; diabetes; cancer; cardiovascular, renal or liver disease; or use of medications expected to affect weight. Randomization to HLF or HLC was performed using an allocation sequence determined by computerized random-number generation [27]. The weight loss intervention involved a 12-month protocol of 22 small-group educational sessions focused on three central components for both HLC and HLF [28]. During the first 8 weeks of limbo phase, participants were instructed to cut back on fat or carbohydrate intake progressively until they achieved a daily intake of no more than 20 g of carbohydrate (HLC) or fat (HLF). During the titrate phase, participants were instructed to increase their fat or carbohydrate intake slowly, by 5–15 g each week, until they achieved a comfortable maintenance level. The goal of this phase was to find the lowest intake of fat or carbohydrates that each participant could maintain for the 12-month intervention period. The third diet intervention component was high quality, for which both groups received similar instructions to focus on home-cooked whole foods, maximize fresh, seasonal vegetables, lean, grass-fed meats, and eliminate or minimize processed foods with added sugar, refined white flour and *trans-*fats. All study participants provided written informed consent about the study procedures. The study was registered on clinicaltrials.gov with the identifier: NCT01826591.

### Outcome measurements

#### Weight, height, body mass index, and fat and lean body mass

Body weight was measured at baseline, and months 3, 6, and 12. Weight was measured in light clothing to the nearest 0.1 kg using a calibrated clinical scale. Height was measured to the nearest 0.1 cm using a Seca wall-mounted stadiometer. Body Mass Index (BMI) was calculated as the weight in kg divided by height in meters squared.

Body composition was assessed by dual-energy x-ray absorptiometry (DEXA) at baseline and months 6 and 12 [28]. Access to this was made available only after the first *n* = 78 participants had been enrolled (i.e., none of the first 78 participants had a baseline measurement taken); from that point on DEXA was assessed in 87% of the remaining participants (*n* = 276 women, *n* = 190 men).

#### Dietary intake and adherence

Dietary intake was assessed at baseline and months 3, 6, and 12 using three unannounced 24-h multiple-pass recall interviews (2 weekdays, 1 weekend day) [29]. Adherence was measured via a weight-adjusted standardized adherence (WASA) score based on the deviation score (DS) between the Limbo phase macronutrient goal (20 g of fat on HLF or carbohydrate on HLC) and participants reported dietary intake. A WASA score was calculated for each participant by diet at each time point (3, 6, or 12 months) with available dietary data as follows: (a) up to three recalls per data collection time point were averaged as an estimate of macronutrient consumption, e.g., 50 g carbohydrates; (b) deviation from the target of 20 g of carbohydrates (or fat) was calculated as 20–50 = −30 g; (c) the DS was equal to the deviation divided by baseline weight in kg, e.g., −30/60 kg yields DS of −1/2; (d) DS were normalized (*Z*-score) within diet and timepoint by subtracting the mean and dividing by the standard deviation, e.g., if the mean (standard deviation) DS of HLC at 3 months was 0.5 [4], the Z-score at 3 months would be (−1/2–0.5)/4 = −1/4; and, (e) WASA for each participant was calculated as the average of available *Z*-scores at 3, 6, and 12 months. A WASA score of 0 reflects an average degree of adherence relative to all groups; positive and negative scores reflect better and worse adherence than average across all groups, respectively. A 12-month dietary WASA for each participant was then calculated as the average of any available WASA scores from the three post-randomization time points (3, 6, and 12 months).

#### Food choice questionnaires

To assess attitudes toward dietary carbohydrates or fat, we selected two questions from the battery of psychosocial questionnaires administered in the study that were adapted from a previously validated food choice questionnaire (FCQ) developed by Steptoe et al.: The first statement asked to express the degree of preference for LF food on a typical day [statement: “It is important to me that the food I eat on a typical day: Is low in Fat”: possible answers: (1) Very important, (2) Moderately important, (3) A little important, (4) Not at all important]. The second statement asked to report the tendency to avoid foods high in refined carbohydrates [statement: “Particularly avoid foods with high carbohydrate content (e.g., bread, pasta, rice, etc.); possible answers: (1) Always, (2) Very Often, (3) Often, (4) Sometimes, (5) Rarely, (6) Never].

### Statistical analysis

We tested three main hypotheses: (i) 12-month changes in key outcomes (weight, fat mass, and lean mass) were different by diet-sex group (HLC women, HLC men, HLF women, HLF men), (ii) adherence was different by diet-sex group, (iii) 12-month changes in key outcomes by diet-sex group were mediated by adherence.

Linear mixed effects models were used to address hypothesis (i). Linear mixed effects can flexibly model incomplete longitudinal data under a missing at random assumption. Models included fixed effects for diet-sex group, time, and their interaction, with additional fixed effects for the potential confounders of baseline weight and baseline percent fat, and included a random intercept term for participants. An *F*-test with Satterthwaite adjustment for denominator degrees of freedom was used to test the null hypothesis that 12-month changes are equivalent in diet-sex groups. T-tests with Kenward-Roger degrees of freedom adjustment were employed for the four pairwise comparisons of interest.

To address hypothesis (ii), WASA scores were modeled in a linear regression as a function of diet-sex group. An overall *F*-test was used to test the association, with *t*-tests for four pairwise comparisons of interest: HLC vs. HLF within women; HLC vs. HLF within men; men vs. women within HLF; and, men vs. women within HLC.

Hypothesis (iii) was tested using the same linear mixed model framework as hypothesis (i), with the addition of WASA as a fixed effect. Overall *F*-tests and pairwise *t*-tests (both with appropriate degrees of freedom adjustment) were again employed to test differences in 12-month outcomes. Relationships between adherence (WASA) and percent change in 12-month outcomes were also characterized via scatterplot and Spearman rank correlation.

All statistical tests were two-sided at significance level 0.05. Given the exploratory nature of this secondary analysis, no adjustments for multiple testing were performed. All statistical analyses were carried out using R version 3.6.1 [30] and code is available at https://github.com/joerigdon/DIETFITS_Gender.

## Results

### Baseline characteristics of the study population

Among 609 participants originally randomized to HLC (women, *n* = 179; men, *n* = 125) or HLF (women, *n* = 167; men, *n* = 138), overall 12-months retention, i.e., providing any data at 12 months, was ~79% for each group, with no significant between-group differences. Mean age was about 38–40 years for all four groups. There were no statistically significant baseline differences in weight between HLC and HLF women or between HLC and HLF men (Table 1). Women weighed less than men, but all groups had similar BMI (about 33 kg/m^2^). Participants who received DEXA measurement (*n* = 466) were heavier (5 ± 1.4 kg) than those who did not (*n* = 143) (Supplementary Table S1). Men had a significantly higher MetS score and associated variables (i.e., triglycerides, HDL-C, insulin-30, fasting glucose and insulin, blood pressure) compared with women, which is in line with their physiological propensity to store fat in visceral rather than subcutaneous depots.

**Table 1:** Baseline demographics and anthropometric and metabolic variables.

	HLC women	HLC men	HLF women	HLF men	*P* value^1^
	*n* = 179	*n* = 125	*n* = 167	*n* = 138	
Age (years)	40.2 (±6.9)	40.2 (±6.5)	39.7 (±6.4)	38.9 (±7.3)	0.41
Education					
High school	48 (26.8%)	34 (27.2%)	41 (24.6%)	29 (21.0%)	0.85
College	73 (40.8%)	45 (36.0%)	68 (40.7%)	59 (42.8%)	
Grad degree	58 (32.4%)	45 (36.0%)	57 (34.1%)	50 (36.2%)	
Missing	0 (0.0%)	1 (0.8%)	1 (0.6%)	0 (0.0%)	
Race/ethnicity					
White	111 (62.0%)	71 (56.8%)	91 (54.5%)	85 (61.6%)	0.068
Hispanic	35 (19.6%)	26 (20.8%)	44 (26.3%)	23 (16.7%)	
Asian	19 (10.6%)	11 (8.8%)	9 (5.4%)	21 (15.2%)	
African-American	7 (3.9%)	6 (4.8%)	8 (4.8%)	2 (1.4%)	
AI/AN/PI	0 (0.0%)	0 (0.0%)	2 (1.2%)	1 (0.7%)	
Other	7 (3.9%)	11 (8.8%)	13 (7.8%)	6 (4.3%)	
Weight (kg)	88.9 (±12.5)^ab^	106.8 (±13.7)^ac^	90.7 (±11.5)^cd^	105.7 (±13.9)^bd^	<0.0001
BMI (kg/m^2^)	32.9 (±3.4)	33.8 (±3.4)	33.3 (±3.4)	33.5 (±3.4)	0.16
Body fat (%)	40.4 (±4.0)^ab^	30.3 (±4.7)^ac^	41.0 (±3.9)^cd^	29.9 (±4.5)^bd^	<0.0001
Missing	34 (19.0%)	32 (25.6%)	36 (21.6%)	41 (29.7%)	
Waist circumference (cm)	102.6 (±10.5)^ab^	112.7 (±9.9)^ac^	103.5 (±10.4)^cd^	111.8 (±9.7)^bd^	<0.0001
Missing	0 (0%)	2 (1.6%)	1 (0.6%)	2 (1.4%)	
LDL (mg/dL)	111.6 (±26.3)	117.1 (±25.6)	109.1 (±29.0)	114.8 (±32.1)	0.056
Missing	1 (0.6%)	0 (0%)	0 (0%)	0 (0%)	
HDL (mg/dL)	52.3 (±9.4)^ab^	46.1 (±7.6)^ac^	52.1 (±9.1)^cd^	46.2 (±7.4)^bd^	<0.0001
Triglycerides (mg/dL)	117.3 (±104.2) ^ab^	143.4 (±66.3)	114.9 (±66.0)^cd^	145.2 (±74.0)^bd^	<0.0001
SBP (mmHg)	120.2 (±12.6)^ab^	126.7 (±11.0)^ac^	118.6 (±11.9)^cd^	127.9 (±11.4)^bd^	<0.0001
Missing	1 (0.6%)	0 (0%)	2 (1.2%)	0 (0%)	
DBP	79.7 (±7.8)^ab^	83.3 (±7.3)^ac^	78.8 (±6.9)^cd^	83.6 (±7.0)^bd^	<0.0001
Missing	1 (0.6%)	0 (0%)	2 (1.2%)	0 (0%)	
Fasting glucose (mg/dL)	96.5 (±9.0)^ab^	101.2 (±10.1)^ac^	96.8 (±8.3)^cd^	100.9 (±8.5)^bd^	<0.0001
Fasting insulin (μIU/mL)	14.2 (±7.3)^ab^	17.2 (±8.7)	13.6 (±6.7)^cd^	18.6 (±18.3)^bd^	<0.0001
Insulin-30 (μIU/mL)	85.1 (±57.8)^ab^	101.4 (±66.0)^a^	88.5 (±66.5)	103.1 (±68.1)^b^	0.005
Missing	0 (0%)	0 (0%)	2 (1.2%)	1 (0.7%)	
Metabolic syndrome					
No	121 (67.6%)^ab^	47 (37.6%)^ac^	110 (65.9%)^cd^	52 (37.7%)^bd^	<0.0001
Yes	58 (32.4%)	78 (62.4%)	57 (34.1%)	86 (62.3%)	
Respiratory exchange ratio	0.86 (±0.06)	0.87 (±0.06)	0.85 (±0.06)	0.87 (±0.07)	0.087
Missing	25 (14.0%)	15 (12.0%)	21 (12.6%)	20 (14.5%)	
Resting energy expenditure (kcal)	1488.2 (±215.4)^ab^	1825.3 (±275.0)^ac^	1500.8 (±213.6)^cd^	1835.6 (±245.8)^bd^	<0.0001
Missing	25 (14.0%)	15 (12.0%)	21 (12.6%)	20 (14.5%)	
Energy expenditure (kcal/kg/d)	32.5 (±2.9)	32.8 (±2.6)	32.7 (±1.7)	32.5 (±1.7)	0.56
Missing	26 (14.5%)	6 (4.8%)	17 (10.2%)	6 (4.3%)	
Genotype					
Low-carb	57 (31.8%)	40 (32.0%)	40 (24.0%)	43 (31.2%)	0.15
Low-fat	69 (38.5%)	45 (36.0%)	75 (44.9%)	55 (39.9%)	
Neither	40 (22.3%)	36 (28.8%)	42 (25.1%)	28 (20.3%)	
None	3 (1.7%)	0 (0.0%)	6 (3.6%)	6 (4.3%)	
Missing	10 (5.6%)	4 (3.2%)	4 (2.4%)	6 (4.3%)	
Smoking status					0.22
Non-Smoker	137 (76.5%)	92 (73.6%)	137 (82.0%)	101 (73.2%)	
Current smoker	0 (0.0%)	0 (0.0%)	0 (0.0%)	0 (0.0%)	
Past smoker	42 (23.5%)	33 (26.4%)	30 (18.0%)	37 (26.8%)	

*AI* American Indian, *AN* Alaskan Native, *PI* Pacific Islander.

^1^Shared superscript symbols (a, b, c, d, e) indicate significant between-group differences (Kruskal–Wallis test for continuous variables, e.g., age, and Fisher’s exact test for categorical variables, e.g., race).

There was no significant between-group difference in baseline percent macronutrient intake between groups with the exception of a modest 1% greater intake of protein among HLC men compared to HLF men (*P* = 0.047) (Table 2). At all postrandomization time points, HLC women and men reported lower percent carbohydrate intake than HLF, and HLF women and men reported lower percent fat intake than HLC (*P* < 0.001).

**Table 2:** Macronutrient intake by subgroup and time point.

	HLC women	HLC men	HLF women	HLF men^1^	*P* value^2^
Total calories, mean (SD)					
Baseline	2096 (±615)^ab^	2405 (±665)^ac^	1985 (±605)^cd^	2345 (±729)^bd^	<0.0001
3 months	1492 (±440)^ab^	1708 (±512)^ac^	1410 (±380)^cd^	1648 (±514)^bd^	<0.0001
6 months	1508 (±429)^ab^	1779 (±605)^ac^	1429 (±421)^cd^	1872 (±651)^bd^	<0.0001
12 months	1576 (±442)^ab^	1871 (±483)^ac^	1587 (±452)^cd^	1884 (±551)^bd^	<0.0001
Total carb, mean (SD), g					
Baseline	237 (±76)^a^	261 (±80)^ab^	228 (±76)^bc^	259 (±95)^c^	0.001
3 months	99 (±58)^ab^	93 (±55)^cd^	189 (±61)^ace^	226 (±78)^bde^	<0.0001
6 months	108 (±56)^ab^	120 (±75)^cd^	185 (±63)^ace^	244 (±91)^bde^	<0.0001
12 months	129 (±62)^ab^	137 (±66)^cd^	195 (±68)^ace^	236 (±78)^bde^	<0.0001
Total fat, mean (SD), g					
Baseline	88 (±32)^a^	100 (±35)^ab^	79 (±29)^bc^	96 (±39)^c^	<0.0001
3 months	83 (±27)^ab^	98 (±37)^cd^	41 (±18)^ac^	44 (±23)^bd^	<0.0001
6 months	81 (±27)^ab^	95 (±35)^cd^	45 (±21)^ace^	57 (±32)^bde^	<0.0001
12 months	79 (±27)^ab^	96 (±32)^cd^	54 (±22)^ace^	61 (±28)^bde^	<0.0001
Total protein, mean (SD), g					
Baseline	86 (±25)^ab^	104 (±30)^ac^	84 (±25)^cd^	102 (±31)^bd^	<0.0001
3 months	87 (±24)^ab^	110 (±38)^acd^	73 (±20)^bce^	88 (±32)^de^	<0.0001
6 months	85 (±24)^ab^	106 (±34)^acd^	72 (±23)^bce^	94 (±32)^de^	<0.0001
12 months	85 (±22)^a^	106 (±35)^ab^	78 (±25)^bc^	93 (±27)^c^	<0.0001
Total carb, mean (SD), %					
Baseline	45 (±7)	43 (±7)	45 (±7)	44 (±10)	0.15
3 months	25 (±11)^ab^	21 (±11)^cd^	52 (±10)^ac^	53 (±11)^bd^	<0.0001
6 months	27 (±10)^ab^	26 (±11)^cd^	51 (±10)^ac^	51 (±11)^bd^	<0.0001
12 months	31 (±11)^ab^	28 (±11)^cd^	48 (±10)^ac^	49 (±9)^bd^	<0.0001
Total fat, mean (SD), %					
Baseline	36 (±6)	36 (±6)	35 (±6)	35 (±7)	0.095
3 months	49 (±9)^ab^	50 (±9)^cd^	25 (±8)^ac^	23 (±8)^bd^	<0.0001
6 months	47 (±9)^ab^	46 (±9)^cd^	27 (±8)^ac^	26 (±9)^bd^	<0.0001
12 months	44 (±9)^ab^	45 (±9)^cd^	30 (±8)^ac^	28 (±7)^bd^	<0.0001
Total protein, mean (SD), %					
Baseline	17 (±4)^a^	18 (±3)^a^	18 (±4)	18 (±5)	0.047
3 months	25 (±6)^ab^	27 (±7)^cd^	21 (±5)^ac^	22 (±7)^bd^	<0.0001
6 months	24 (±6)^ab^	25 (±7)^cd^	21 (±6)^ac^	21 (±7)^bd^	<0.0001
12 months	23 (±6)	23 (±6)^bc^	21 (±6)^b^	21 (±6)^c^	0.0007

^a^Dietary data were available at baseline and 3, 6, and 12 months, respectively, as follows: HLC women, *n* = 179, 162, 146, 132; HLC men, *n* = 125, 113, 105, 92; HLF women, *n* = 166, 153, 134, 127; HLF men, *n* = 138, 121, 106, 98.

^b^From Kruskal–Wallis omnibus test; shared superscript symbols (a, b, c, d, e) indicate significant between-group differences (Dunn’s test, Bonferroni adjusted *p* < 0.05).

### Changes in weight, body fat, and lean mass

**Fig. 1:**
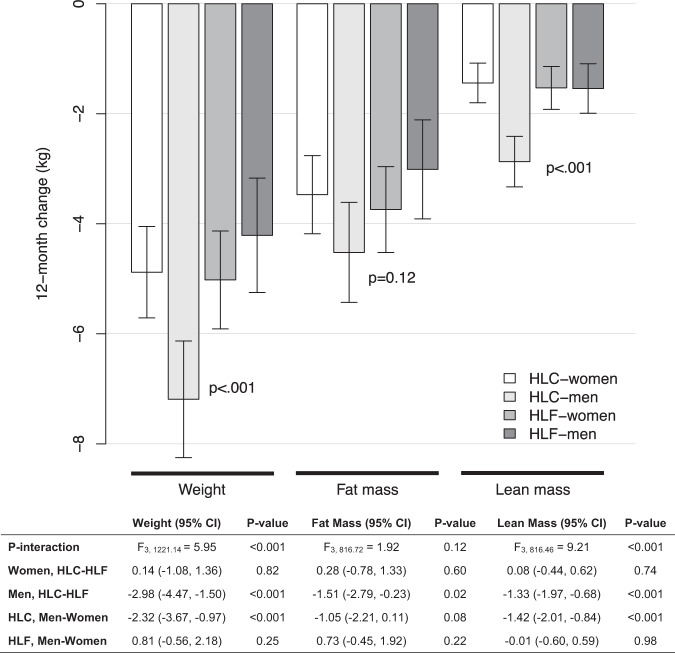
12-month changes in weight, fat mass, and lean mass by sex and diet, adjusted for baseline weight and baseline body fat percentage. Estimated between-group differences (95% CI). Weight data were available at baseline and 3, 6, and 12 months, respectively, as follows: women on HLF, *n* = 167, 151, 131, 120; women on HLC, *n* = 179, 155, 144, 131; men on HLF, *n* = 138, 119, 97, 96; and men on HLC, *n* = 125, 108, 95, 89. Fat mass and lean mass data were available at baseline, 6, 12 months, respectively, as follows (cohorts 2–5): women on HLF, *n* = 131, 107, 106; women on HLC, *n* = 145, 121, 118; men on HLF, *n* = 97, 75, 79; men on HLC, *n* = 93, 73, 78.

After adjustment for differences in baseline weight and body fat percentage, we observed significant differences in 12-month weight loss [*F*(3, 1221.14) = 5.95, *P* < 0.001] and loss of lean mass [*F*(3, 816.46) = 9.21, *P* < 0.001]. Among men, HLC induced significantly greater weight loss than HLF [−2.98 kg (−4.47, −1.50); *P* < 0.001]. This was also observed for fat mass [−1.51 (−2.79, −0.23); *P* = 0.02], and, for lean mass [−1.33 (−1.97, −0.68); *P* < 0.001] (Fig. 1). In contrast, there was no differential effect by diet type on weight loss, and loss of fat and lean mass among women, who achieved similar changes on HLC and HLF. In addition, HLC men lost significantly more weight [−2.32 (−3.67, −0.97); *P* < 0.001] and lean mass [−1.42 (−2.01, −0.84); *P* < 0.001] than HLC women. The loss of weight, fat mass, and lean mass were not significantly different between HLF women and HLF men.

### Diet adherence

**Fig. 2:**
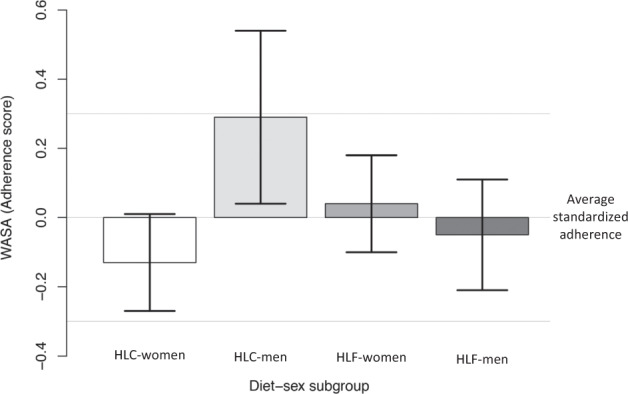
Differences in 12-month diet adherence by sex and diet type. Estimated between-group differences in weight-adjusted standardized adherence (WASA) scores. Higher values indicate higher adherence relative to the average adherence across all groups.

Though the overall differences in adherence by diet-sex group were not statistically significant [*F*(3, 553) = 2.06, *p* = 0.10] (Fig. 2), HLC men and HLC women were the most and least adherent of all groups, respectively; in pairwise analyses this difference reached modest significance [WASA difference: 0.25 (0.04, 0.46); *P* = 0.02].

### Associations between diet adherence and changes in weight, body fat, and lean mass

For all groups diet adherence correlated significantly with 12-month changes in percent weight (HLC women, rs = −0.36, *P* < 0.001; HLC men, rs = −0.57, *P* < 0.001; HLF women, rs = −0.32, *P* < 0.001; HLF men, rs = −0.36, *P* < 0.001) and fat mass with the exception of HLF men (HLC women, rs = −0.29, *P* = 0.002; HLC men, rs = −0.60, *P* < 0.001; HLF women, rs = −0.27, *P* = 0.01; HLF men, rs = −0.22, *P* = 0.066) (Supplementary Figs. S1 and S2). Diet adherence also correlated significantly with 12-month percent changes in lean mass for all the groups with the exception of HLF women bordering on the *P* < 0.05 significance cutoff (HLC women, rs = −0.23, *P* = 0.014; HLC men, rs = −0.45, *P* < 0.001; HLF women, rs = −0.20, *P* = 0.054; HLF men, rs = −0.29, *P* = 0.016) (Supplementary Fig. S3). Weight loss estimates within group remained similar after adjusting for adherence, suggesting it was not a mediator (Supplementary Table S2).

### Attitudes toward dietary fats and carbohydrates

The food choice questionnaire data were used to explore whether the observed differences in diet adherence between women and men might reflect different attitudes toward dietary fats and carbohydrates (Table 3). At baseline, a significantly greater proportion of women vs. men (58% vs. 39%, *p* ≤ 0.0001) rated the importance of consuming LF foods as very or moderately important. Also assessed at baseline were attitudes toward foods particularly high in refined carbohydrates (e.g., bread, pasta, rice), which were restricted on both HLC and HLF. A significantly greater proportion of women vs. men (27% vs. 18%, *p* = 0.0009) reported a tendency to avoid these foods *always, very often* or *often*.

**Table 3:** Food attitudes at baseline, by sex.

	Female	Male	*P* value^a^
	*n* = 346	*n* = 263	
Low-fat food on a typical day			
Very important	45 (13.0%)	23 (8.7%)	<0.0001
Moderately important	157 (45.4%)	79 (30.0%)	
A little important	107 (30.9%)	115 (43.7%)	
Not at all	32 (9.2%)	43 (16.3%)	
Missing	5 (1.4%)	3 (1.1%)	
Avoiding refined carbohydrates			
Always	4 (1.2%)	3 (1.1%)	0.0009
Very often	43 (12.4%)	21 (8.0%)	
Often	48 (13.9%)	24 (9.1%)	
Sometimes	103 (29.8%)	55 (20.9%)	
Rarely	89 (25.7%)	97 (36.9%)	
Never	54 (15.6%)	59 (22.4%)	
Missing	5 (1.4%)	4 (1.5%)	

^a^Fisher’s exact test.

## Discussion

By factoring sex into this secondary analysis of the DIETFITS trial, we identified a significant interaction of sex on the effects of a HLC vs. HLF diet on weight loss success, that was not addressed in the parent analysis, when data on women and men were combined [27]. After accounting for differences in baseline weight and body fat percentage, we observed a significant effect of diet intervention type on weight, fat, and lean loss among men but not among women. Men lost significantly more weight, fat mass, and lean mass on HLC diet vs. HLF, whereas women achieved similar losses on HLC vs. HLF. In addition, within the HLC group, men lost significantly more weight than women, which was not true for HLF.

Behavioral differences in diet adherence may explain the greater weight loss success of men on HLC. We found that men were significantly more adherent to HLC than women. Interestingly, prior to randomization, the women expressed a significantly greater preference for LF foods than the men, which might have made it more difficult for the former to adhere to the higher fat content of HLC. In line with an overall greater attitude to weight control, the women also reported a significantly higher tendency to avoid foods high in refined carbohydrates (e.g., bread, pasta, rice). However, since these foods were restricted on both HLC and HLF, this attitude should not have differentially affected diet adherence to HLC or HLF among women. These findings are consistent with several large population studies indicating that women express a greater preference for LF products and a greater concern toward high-fat foods than men [12, 31–41]. Sociologists suggest that women may be concerned about eating dietary fats due to a gender stereotype that pressure them to be slim [42–44] and avoid foods perceived as “fattening” [45–47]—a stereotype that is reflected and reinforced by gender marketing of LF products to women [48, 49].

Although adherence was significantly correlated with weight loss for all groups, this association was of greater magnitude for HLC than HLF. This is in agreement with a previous study that observed a significant association between greater adherence to a low carb diet and greater weight loss that was not observed for the low-fat diet [26]. The association between diet adherence and weight loss was also greater for men than for women, with HLC men having the strongest correlation.

Our findings are consistent with previous reports that a low carb diet may produce more effective weight loss in men than women [9, 16, 17]. Volek et al. reported that men (*n* = 13) lost significantly more absolute weight and fat on a very low-carbohydrate diet (VLCD) compared with a LF diet, whereas VLCD was less effective for women (*n* = 15) [9]. However, comparisons between women and men on either VLCD or LF were not made, probably due to the small sample size. Similarly, in a study of 33 men and 45 women on four dietary regimens (Atkins, Slim-Fast, Weight-Watchers, and Rosemary Conley’s Diet), Millward et al. reported that men in the Atkins group had a significantly greater, albeit transient, reduction in body weight and fat compared to all other groups. However, baseline weight was not accounted. In a 2-year study of 322 moderately obese participants (men: 86%), Shai et al. found that a low-carbohydrate diet was more effective for men whereas women tended to lose more weight on a Mediterranean diet [16]. The current study builds on these previous studies and identifies a significant effect modification of sex on both weight loss and loss of lean mass. Not only did HLC men lost significantly more weight than HLF men, they also lost both more fat and lean mass, with the lean mass differences being more statistically significant than the fat mass differences.

Our analysis has several important strengths. First, we used data derived from a relatively large RCT with sample sizes in each of the four diet-sex groups of *n* = 125 to 179, good retention (~70% for all groups), and a sufficient duration (12 months) to evaluate long-term effects of two diets with substantially different macronutrient composition. In addition, we designed a novel WASA score to enable comparisons across diet and sex groups.

This study has also several limitations. First, this secondary analysis was not set out in the original study protocol, and hence participants were not stratified by sex prior to randomization. Nonetheless, randomization yielded similar baseline characteristics within each sex. A second major limitation is that we did not analyze other sex-related factors, such as genotype, hormones, MetS, or psychosocial factors that might affect either diet adherence or weight loss response. Third, although our WASA score was specifically designed to enable comparisons across both sex and diet, this novel metric has not been validated. Therefore, as any comparison of data between groups with different means, ranges, and standard deviations, it should be interpreted with caution. Self-report of dietary intake might also have affected the assessment of adherence. For example, individuals who underreported their intake—as is common in diet assessment [50]—might have been misclassified with an inaccurate WASA. Finally, as in most weight loss diet studies, complete data were missing for a subset of participants at both baseline and 12 months.

In conclusion, we found modest but significantly different losses of weight, fat mass, and lean mass by diet-sex groups, with adherence being significantly correlated with weight loss in all groups. Women may find it easier to adhere to a LF diet than to a low-carb diet due to gender norms and marketing strategies that make LF products more appealing and accessible in the marketplace. To increase low-carb adherence, and thus increase opportunity for success, healthcare providers may want to inform their female patients about the comparable effectiveness of a low-carb diet to LF diet, as such beliefs have a strong influence of food attitudes [51–54]. Our findings also beg the question of whether heterogeneous treatment effects (HTEs) due to sex differences could explain some of the variability in weight loss outcome in response to different diet interventions. Since any subgroup analysis lessens power and therefore the ability to detect effects, this question should be tested in randomized clinical trials with large sample sizes and stratification by sex prior to randomization. Unbiased estimation of sex-related HTEs on diet response from such a trial could provide better clinical evidence for the implementation of personalized weight loss strategies based on sex differences. As precision medicine grows steadily, analysis of sex differences should become a priority in comparative effectiveness trials of different diets designed for healthy weight loss [55, 56].

## Supplementary information

Table S1. Baseline Demographics and Anthropometric Variables of Subjects with and without DEXA Measurement

Figure S1

Figure S2

Figure S3

supplementary legends
