# Supplementary material for: Examining differences between overweight women and men in 12-month weight loss study comparing healthy low-carbohydrate vs. low-fat diets
Source: Int J Obes (Lond). 2020 Nov 14;45(1):225–34. doi: 10.1038/s41366-020-00708-y (PMC7752762; doi:10.1038/s41366-020-00708-y)
Supplement: Supplementary file 1 — Table S1. Baseline Demographics and Anthropometric Variables of Subjects with and without DEXA Measurement [file 41366_2020_708_MOESM1_ESM.docx]

**Table S1. Baseline Demographics and Anthropometric Variables of Subjects with and without DEXA Measurement (n=609)**

|  | **DXA measured** | **DXA missing** | **P-value^a^** |
| --- | --- | --- | --- |
|  | n=466 | n=143 |  |
| **Age, mean (SD), years** | 39.1 (±6.7) | 40.0 (±6.9) | 0.13 |
| **Weight, mean (SD), kg** | 95.7 (±14.6) | 100.7 (±16.5) | 0.002 |
| **Body Mass Index, mean (SD), kg/m^2^** | 33.2 (±3.3) | 33.9 (±3.4) | 0.041 |

^a^Wilcoxon rank-sum test
