# Supplementary figures and images for: Examining differences between overweight women and men in 12-month weight loss study comparing healthy low-carbohydrate vs. low-fat diets
Source: Int J Obes (Lond). 2020 Nov 14;45(1):225–34. doi: 10.1038/s41366-020-00708-y (PMC7752762; doi:10.1038/s41366-020-00708-y)

**HLC-women**

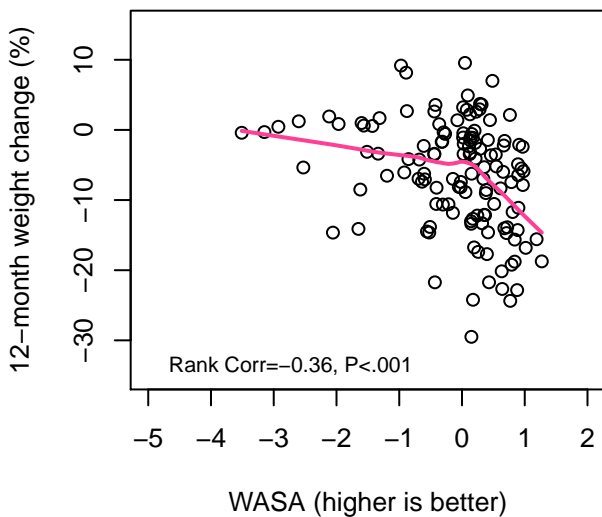

**HLC-men**

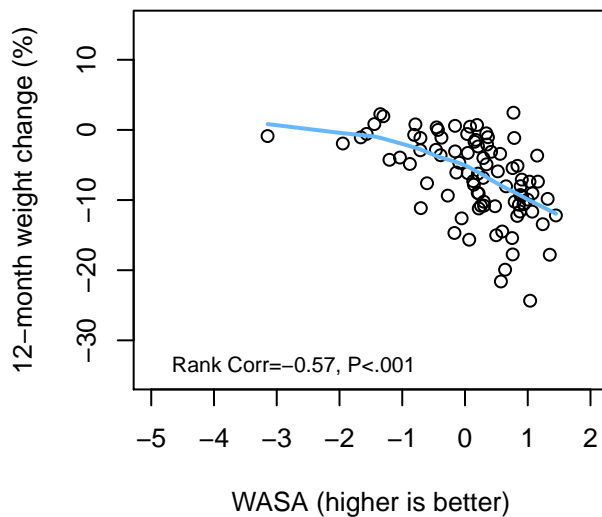

**HLF-women**

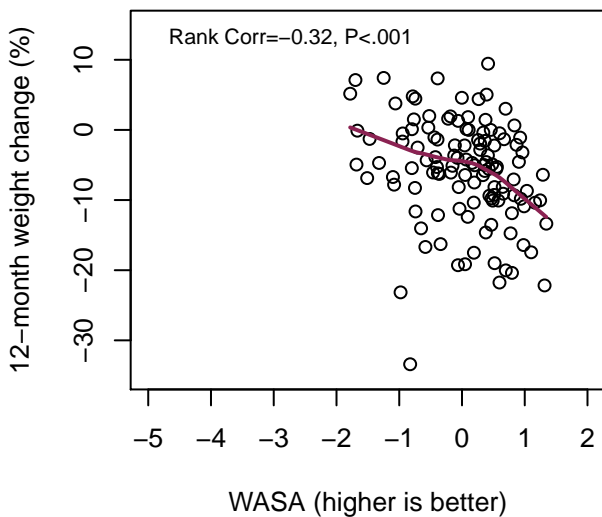

**HLF-men**

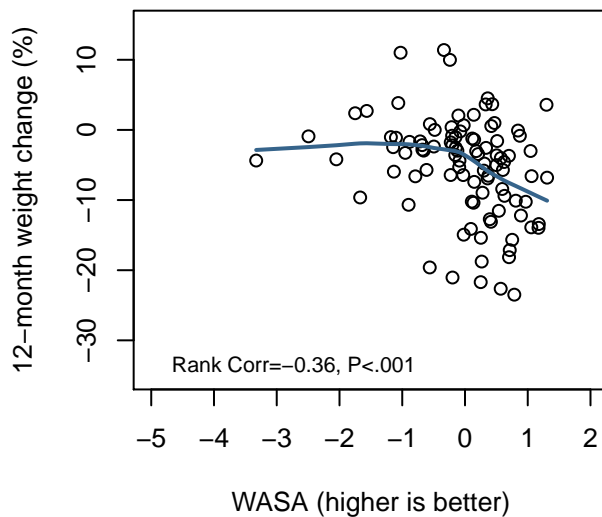

Supplement: Supplementary file 2 — Figure S1 [file 41366_2020_708_MOESM2_ESM.pdf]

**HLC-women**

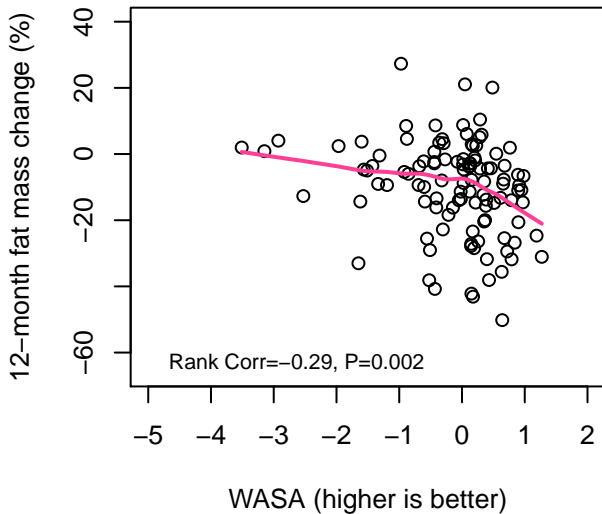

**HLC-men**

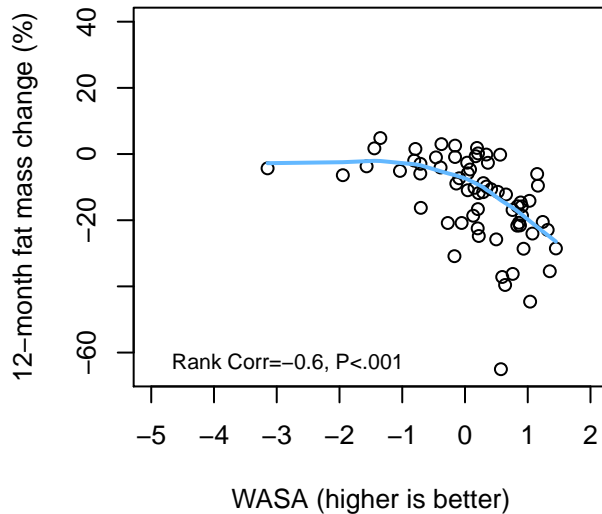

**HLF-women**

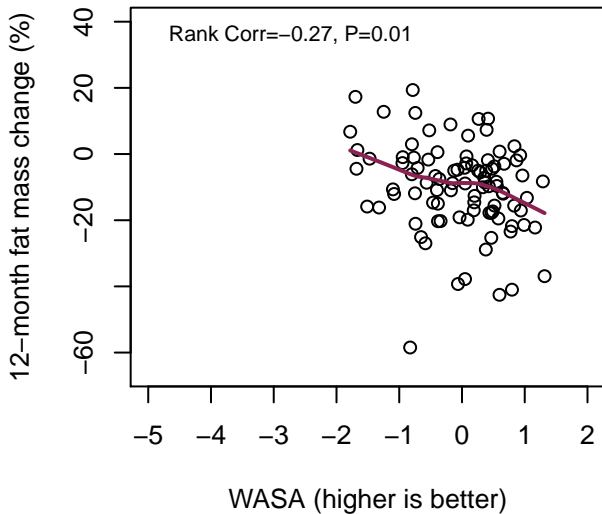

**HLF-men**

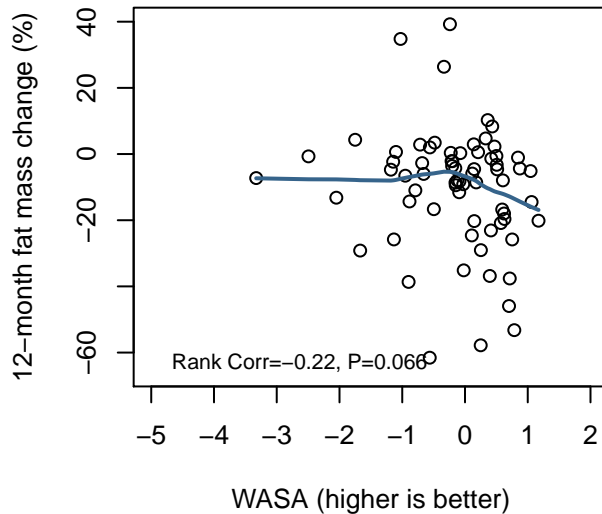

Supplement: Supplementary file 3 — Figure S2 [file 41366_2020_708_MOESM3_ESM.pdf]

**HLC-women**

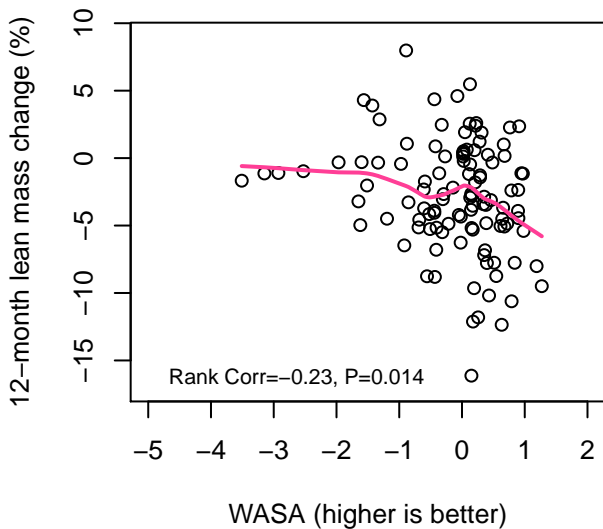

**HLC-men**

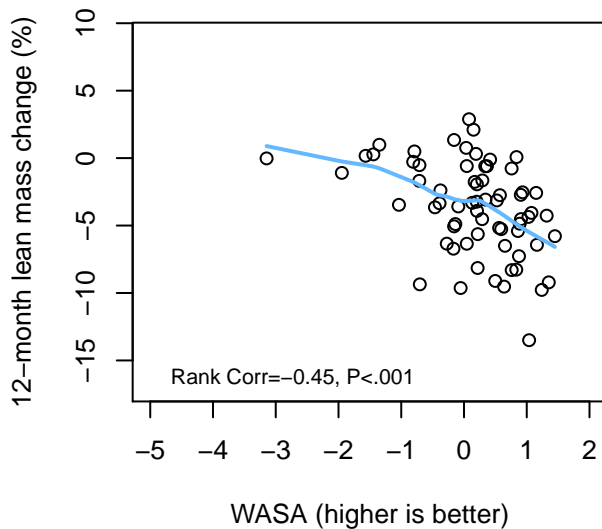

**HLF-women**

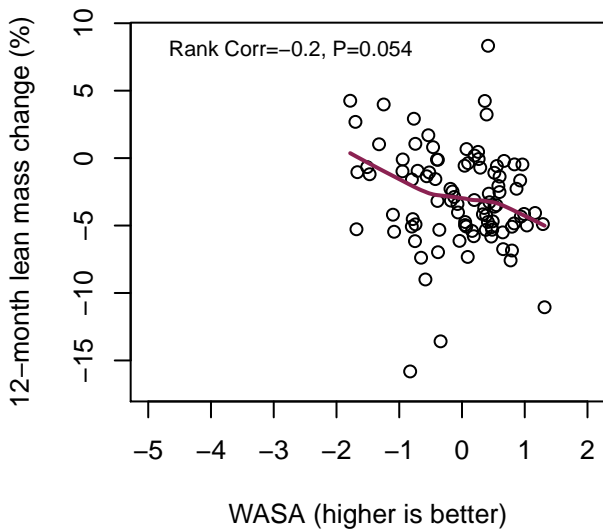

**HLF-men**

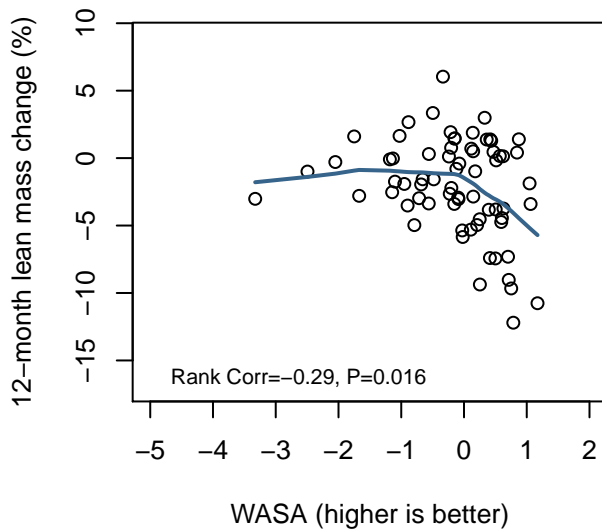

Supplement: Supplementary file 4 — Figure S3 [file 41366_2020_708_MOESM4_ESM.pdf]
