# Supplementary material for: Examining differences between overweight women and men in 12-month weight loss study comparing healthy low-carbohydrate vs. low-fat diets
Source: Int J Obes (Lond). 2020 Nov 14;45(1):225–34. doi: 10.1038/s41366-020-00708-y (PMC7752762; doi:10.1038/s41366-020-00708-y)
Supplement: Supplementary file 5 — supplementary legends [file 41366_2020_708_MOESM5_ESM.docx]

**Figure S1. Association between diet adherence and 12-month percent weight changes**

^a^ Measured as percent change from baseline value for all participants with available data at both baseline and 12 months: HLC-women, N=131 (73.2%), HLC-men, N=89 (71.2%), HLF-women, N=120 (71.9%); HLF-men, N=96 (69.6%)

^b^ rs: Spearman's rank correlation coefficient.

^c^ WASA: Weight-adjusted standardized adherence. A higher WASA score indicates higher diet adherence relative to the average adherence across all groups.

**Figure S2. Association between diet adherence and 12-month percent fat mass changes**

^a^ Measured as percent change from baseline value for all participants with available data at both baseline and 12 months: HLC-women, N=110 (61.5%), HLC-men, N=67 (53.6%), HLF-women, N=93 (55.7%); HLF-men, N=69 (50.0%)

^b^ rs: Spearman's rank correlation coefficient.

^c^ WASA: Weight-adjusted standardized adherence. A higher WASA score indicates higher diet adherence relative to the average adherence across all groups.

**Figure S3. Association between diet adherence and 12-month percent lean mass changes**

^a^ Measured as percent change from baseline value for all participants with available data at both baseline and 12 months: HLC-women, N=110 (61.5%), HLC-men, N=67 (53.6%), HLF-women, N=93 (55.7%); HLF-men, N=69 (50.0%)

^b^ rs: Spearman's rank correlation coefficient.

^c^ WASA: Weight-adjusted standardized adherence. A higher WASA score indicates higher diet adherence relative to the average adherence across all groups.
